# Reply to: Galloyl‐bis‐hexahydroxydiphenoyl glucose and endothelial remodelling: Nutrigenomic insights into blackberry's superior vasorelaxant efficacy over mugwort

**DOI:** 10.1113/EP092989

**Published:** 2025-06-18

**Authors:** Afaf Mehiou, Anca Lucau‐Danila, Zachee L. E. Akissi, Chaimae Alla, Nourelhouda Bouanani, Abdelkhaleq Legssyer, Jean‐Louis Hilbert, Sevser Sahpaz, Abderrahim Ziyyat

**Affiliations:** ^1^ Laboratory of Bioresources, Biotechnologies, Ethnopharmacology and Health, Department of Biology, Faculty of Sciences University Mohammed First Oujda Morocco; ^2^ BioEcoAgro Joint Cross‐Border Research Unit, UMRt 1158 Univ. Lille, INRAE, Univ. Liège, UPJV, JUNIA, Univ. Artois, Univ. Littoral Côte d'Opale Villeneuve d'Ascq France; ^3^ Joint Laboratory CHIC41H University of Lille‐Florimond Desprez Villeneuve d'Ascq France

**Keywords:** *Artemisia campestris*, bioactive compounds, gene expression, hypotension, *Rubus ulmifolius*, vasodilatation

1

We would like to thank the authors of the letter (Tao et al., 2025) for their appreciation of our work (Mehiou et al., 2025). They highlighted the value of the synergy between ethnopharmacology and modern omics technologies, emphasizing the importance of experimental physiology in understanding the mechanisms underlying traditional phytotherapy. We also thank them for their thoughtful and constructive critique.

In response to their suggestions, we would like to offer a few arguments and perspectives.

The cited reference (Kalucka et al., 2020) indeed represents an excellent example of targeted transcriptomic analysis at the cellular level, allowing for the identification of tissue‐type‐dependent gene expression patterns through cross‐marker analysis. Our own transcriptomic approach was different in nature, as it focused on the ingestion of a specific product, which by necessity involves passage through the digestive system. Studies comparing gene expression profiles across various organs (such as the liver, muscle, adipose tissue, intestine and brain) following nutritional events have demonstrated that the liver exhibits earlier and more significant transcriptional alterations than other tissues (Saito et al., 2021). Furthermore, the liver has been consistently recognized as one of the most responsive tissues to nutrigenomic stimuli, owing to its central role in nutrient metabolism and energy homeostasis (De Santis et al., 2015; Rosso & Tiribelli, 2024). Given that our study was nutrigenomic in nature and aimed to provide a broad overview – not limited solely to cardiovascular effects – of the impact of these plants on gene expression, we chose to analyse hepatic tissue. This approach allowed us to uncover not only cardiovascular effects but also antiproliferative, antimicrobial, anti‐inflammatory and appetite‐regulating activities, thereby opening multiple avenues for future research.

We fully agree that targeted analysis at the level of specific cell types would provide further, more localized validation of these effects. Based on the directions observed in our data (Mehiou et al., 2025), we are now well‐positioned to pursue follow‐up studies aimed at identifying the cell types most prominently involved in each observed effect. As for the vascular effect – which is the central focus of our study – it is clear that future investigations will focus on vascular tissue and its cellular subtypes.

The comment regarding phytochemical tracking is also entirely relevant. Integrating such an approach could indeed refine our findings and provide a more nuanced validation of the observed effects, ultimately supporting clinical applications.

## AUTHOR CONTRIBUTIONS

Afaf Mehiou: Investigation, formal analysis, data curation, resources, writing—review and editing. Anca Lucau‐Danila: Conceptualization, investigation, formal analysis, data curation, methodology, writing—original draft preparation. Zachee L. E. Akissi: Investigation, formal analysis, data curation, methodology, writing—original draft preparation. Chaimae Alla: Investigation, formal analysis, data curation, resources, writing—review and editing. Nourelhouda Bouanani: Resources, writing—review and editing. Abdelkhaleq Legssyer: Resources, writing—review and editing. Jean‐Louis Hilbert: Conceptualization, supervision, writing—review and editing. Sevser Sahpaz: Conceptualization, supervision, project administration, writing—review and editing. Abderrahim Ziyyat: Conceptualization, supervision, funding acquisition, project administration, writing—review and editing. All authors read and approved the final version of the manuscript. They agree to be accountable for all aspects of the work in ensuring that questions related to the accuracy or integrity of any part of the work are appropriately investigated and resolved. All persons designated as authors qualify for authorship, and all those who qualify for authorship are listed.

## CONFLICT OF INTEREST

None declared.

## FUNDING INFORMATION

This work was supported by the CNRST within the framework of the Research Excellence Grants Program (CNRST grants for ALLA Chaimae, MEHIOU Afaf), and by the aromatic and medicinal plants project 2nd PMA 2020/5 (period 2020‐2024) jointly funded by the Ministry of Higher Education, the National Agency for Aromatic and Medicinal Plants of Taounate, and the Presidency of Mohammed First University.
